# Effect of a Heating System Using a Ground Source Geothermal Heat Pump on Production Performance, Energy-Saving and Housing Environment of Pigs

**DOI:** 10.3390/ani10112075

**Published:** 2020-11-09

**Authors:** Hong Seok Mun, Muhammad Ammar Dilawar, Myeong Gil Jeong, Dhanushka Rathnayake, Jun Sung Won, Kwang Woo Park, Sang Ro Lee, Sang Bum Ryu, Chul Ju Yang

**Affiliations:** 1 Department of Animal Science and Technology, Animal Nutrition and Feed Science Laboratory, Sunchon National University, Suncheon 57922, Korea; mhs88828@nate.com (H.S.M.); ammar_dilawar@yahoo.com (M.A.D.); wjdaudrlf13@naver.com (M.G.J.); dhanus871@gmail.com (D.R.); jongmin2301@naver.com (J.S.W.); 2 WP Co., Ltd., Suncheon 58023, Korea; pkw9872@naver.com (K.W.P.); skylife37@naver.com (S.R.L.); 3 Department of Mechanical Convergence Engineering, Hanyang University, Seoul 04763, Korea; fbvod@hanmail.net

**Keywords:** geothermal heat pump, energy consumption, sustainable, coefficient of performance, pig house, noxious gas emission

## Abstract

**Simple Summary:**

A geothermal heat pump (GHP) was installed in a pig house, and production performance, housing environment, energy efficiency, noxious and carbon dioxide (CO_2_) gas emissions, and economics were compared between GHP and the control (conventional heating). The CO_2_ gas emission, usage, and cost of electricity were reduced in the GHP-installed pig house. The GHP also maintained the inside temperature of the pig house more effectively. Furthermore, the concentration of noxious gas (NH_3_) was also lower during the growing and finishing phase in the GHP-installed pig house. Therefore, the results indicate that the GHP system can be used for sustainable pig production and food security as a climate-friendly renewable energy source for livestock.

**Abstract:**

This study examined the effects of a heating system using a ground source geothermal heat pump (GHP). A GHP was installed in a pig house, and a comparative analysis was performed between the GHP and the control (conventional heating system) in terms of the production performance, housing environment, noxious gas emissions, electricity consumption, and economics. The geothermal system performance index, such as the coefficient of performance (COP), inlet, and outlet temperature, were also evaluated. The outflow temperature during each period (weaning, growing, and finishing) was significantly higher than the inflow temperature in all three components of the GHP system. Similarly, the average internal temperature of the GHP-connected pig house was increased (*p* < 0.05) during each period. The carbon dioxide (CO_2_) concentration, electricity usage, and cost of electricity during the 16-week experimental period were reduced significantly in the GHP system relative to the control. The concentrations of ammonia (NH_3_) during the growing and finishing period and the concentrations of formaldehyde during the weaning phase were also lower in the GHP-installed pig house (*p* < 0.05). These results indicate that the GHP system can be used as an environmentally friendly renewable energy source in pig houses for sustainable pig production without harming the growth performance.

## 1. Introduction

For pigs, efficient production performance is affected by the temperature, humidity, heating system, and environment of the pig house. The temperature of the pig house is the most critical parameter in the weaning phase, which must be around 26 °C. A significant part of the total cost associated with swine farming is to maintain the optimal temperature for piglets [1]. In Korea, the outside temperature is below the freezing point during winter. As a result, pigs are exposed to cold stress; hence, heating the pig house is essential.

Air quality is also one of the important variables that include humidity, dust, and harmful gaseous metabolites. Pig farming in Korea has increased greatly; however, with the increasing production, pig farms have become an active contributor to ammonia (NH_3_) and carbon dioxide (CO_2_) emissions into the environment [2]. Some authors [3,4] have reported that the housing environment plays a major role in the amount of NH_3_ emission from animal farm facilities. The main housing factors for NH_3_ emission are the floor type (concrete slats or plastic slats), a bedded system, and a separate area for feeding and resting [5]. NH_3_ is a notorious gas with adverse effects on pig production, welfare, and health. Moreover, NH_3_ released into the atmosphere is the main cause of environmental pollution associated with agriculture and livestock, constituting 95% of anthropogenic emissions [6]. CO_2_ is the most common greenhouse gas, and 32% of this gas is emitted as a result of electricity use for heating [7]. During the cold season, the concentration of CO_2_ increases due to the heating of the swine house using fossil fuels. Hence, there is a strong need in the pork production industry for the utilization of energy resources that can decrease the emissions of harmful gases and are safe for the health of workers and pigs, environmentally friendly, and cost-effective [8].

Energy shortage is a crucial problem throughout the world, and the pressure is increasing on the supply of energy as its consumption is increasing every year. In global energy consumption, more attention is being paid to the use of renewable energy sources [9]. These renewable sources make a significant difference in reducing noxious gas emission, controlling pollution, and enhancing sustainable development [10]. Furthermore, fluctuations in energy prices and regulations to decrease harmful gases have become major challenges to the pig industry for sustainable production. There is a need for interventions on animal housing aimed at decreasing the energy demands of livestock farms and enhancing energy efficiency by introducing renewable energy sources [11]. A geothermal heat pump (GHP) is an efficient and innovative energy technology that utilizes the groundwater or earth’s natural heat storage capacity for cooling and heating. A GHP can supply at least three units of geothermal energy while consuming only one unit of electricity [12]. During the heating mode, a 30–70% reduction in energy consumption can be achieved. GHP improves the quality of the internal air of the animal house by supplying fresh air, and the system does not produce combustion pollutants directly [8,13]. Moreover, a GHP is considered to be the most energy-efficient, environmentally safe, and economically feasible of all of the heating options [14].

Geologically, South Korea has a huge reservoir of geothermal energy resources (GER). Lee et al. [15] reported that if 2% of the GER from the earth surface to a depth of 5 km deep is used, the energy obtained would be equal to 200 times the primary energy consumption in South Korea annually. In particular, for livestock, GER can be used at depths of up to 100 to 500 m to operate a GHP depending on the heat load of the animal house [16]. GHPs have been used globally as a climate-friendly and renewable energy source not only for industrial applications but also for energy production in agricultural and livestock farms for heating and cooling [17,18]. Moreover, equipping a pig house with a heating system using GER will be more economical, effective, and environmentally clean. Despite these potential advantages, few studies have examined the impact of a heating system using a GHP system on the housing environment and production performance of pigs [11]. Therefore, a study was conducted to examine the effects of a GHP system on the production parameters and housing environment of pigs during winter.

## 2. Materials and Methods

### 2.1. Animal Care

This experimental trial was conducted at the study farm of Sunchon National University, Suncheon, South Korea. The experimental procedures and management and care of pigs were approved and reviewed by the Institutional Animal Care and Use Committee (IACUC), Sunchon National University (SCNU IACUC 2019-12).

### 2.2. Animals and Housing

**Figure 1:**
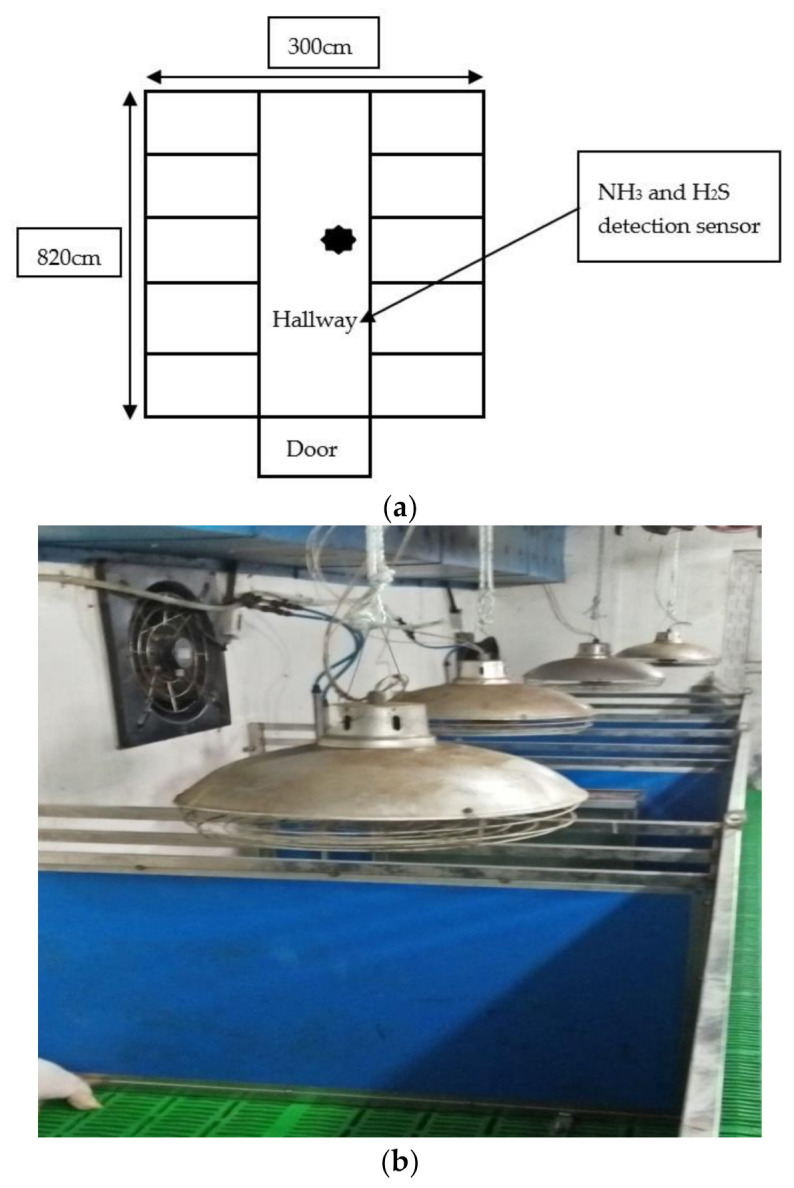
(**a**) Schematic diagram of the pig house with the location of sensors for the detection of noxious gases (NH_3_ and H_2_S); (**b**) inside view of control pig house with electric heating lamps.

**Figure 2:**
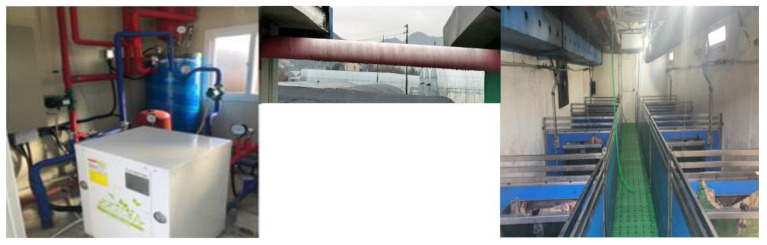
Schematic diagram of the geothermal heat pump (GHP) system for the pig house.

The performance of the GHP was evaluated in winter from 20 December 2019 to 17 January 2020 (four weeks for the weaning period), 18 January to 28 February 2020 (six weeks for the growing period), and 29 February to 10 April 2020 (six weeks for the finishing period). The pigs (Landrace × Duroc × Yorkshire) were reared in two separate rooms (300 cm wide × 820 cm long), which were further divided into ten individual pens (Figure 1a). Out of the two east-facing identical environmentally controlled rooms, the first room was connected to the GHP system (Figure 2), while the other room was installed with a conventional electric heating system (heat lamps) and was used as a control (Figure 1b). The heating lamp (600 W) was placed on the top of each pen, and their height was adjusted according to the age of the pigs.

All pigs were reared in a slatted floor pig house and provided commercially available feed and water ad libitum. The ingredients and chemical composition of the experimental diets are shown in Table 1. The pig houses were well insulated to obtain the desired humidity and temperature during the study trial.

### 2.3. Description of the GHP System

**Figure 3:**
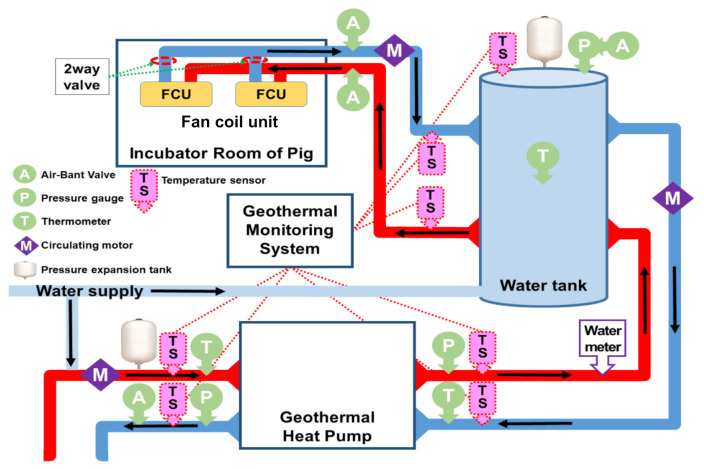
An overview of the ground source geothermal heat pump system.

The GHP system consisted of a borehole exchanger (BHE, double U tube with a depth of 150 m), heating pump unit (HPU), water circulating pumps, fan coil unit (FCU), and a thermal water tank (Figure 3). The water-to-water heat pump unit (Daesung, DHGW 5N-C4-02, Seoul, Republic of Korea) (Supplementary Figure S1) was installed and connected to the pig house using the method reported by Sebarchievici and Sarbu [19]. The energy consumption of the system was 4.93 kW, with a cooling and heating capacity of 20.59 and 19.66 kW, respectively. The water storage capacity of the thermal tank was approximately 260 L and attached to the heat pump to store water for the transfer of heat to the pig house through the FCU. The FCU can provide different wind speeds with temperatures ranging from 5 to 45 °C. The system was equipped with the following three circulating pumps: (1) to transfer groundwater to the heat pump (Model: PH-200M, Wilo pump, Ansan, South Korea, with a flow rate of 136 L/min), (2) to transfer water from the heat pump to the water tank (Model: PH-080M, Wilo pump, Ansan, South Korea, with a flow rate of 75 L/min), and (3) to transfer from the water tank to the pig house (Model: PB-600MA, Wilo pump, Ansan, South Korea, with a flow rate of 80 L/min). Detailed specifications of the geothermal heat pump are presented in Supplementary Table S1.

The HPU consisted of a thermostatic expansion valve, a rolling piston compressor, and a copper tube in tube heat exchangers (evaporator, superheater, and condenser). The condenser can increase the temperature of air from 20 °C to 55–60 °C and distribute it to the pig house through plastic ducts. The air temperature was adjusted in the heat pump by a regulator in response to changes in the pig house temperature. The air within the pig house was displaced by the incoming hot air, which was ventilated by exhaust fans. Detailed specifications of exhaust fans are presented in Supplementary Table S2. The ventilation rate was adjusted at 2 cfu/unit for weaning pigs, 3 cfu/unit for growing pigs, and 7 cfu for finishing pigs. The volumetric flow of air was 700 m^3^/min through the heat pump. Water flows across the tubes while a refrigerant flows inside the tubes in all heat exchangers. The environment friendly working fluid R-410A was used in the heat pump.

### 2.4. Temperature and Coefficient of Performance (COP)

The outside and inside temperature of the control and GHP-installed pig house was measured using temperature detection sensors with a range of −20 to 80 °C ± 0.2 °C and linked with data loggers (CR10X Data Logger, Campbell Scientific, Edmonton, Canada) and integrated negative temperature coefficient thermistor as sensors. The temperature of the cold and hot water (outflow and inflow) of GHP, heating water tank (inflow and outflow), and water storage tank was measured by using a pipe temperature sensor (GPT-1000, Ginice, Korea), which contains a high-quality thermistor sensing element suitable for use in the range from −50 to 150 °C.

The COP of the GHP was calculated using Equations (1) and (2) [8].

Heat absorbed (kW) = M × Cp × ΔT × 4.2 ÷ 3600(1)

(2) $$\mathrm{COP}=\frac{\mathrm{Heat}\;\mathrm{absorbed}\;\left(\mathrm{kW}\right)+\mathrm{Power}\;\mathrm{consumption}\;(\mathrm{kW})\;}{\mathrm{Power}\;\mathrm{consumption}\;(\mathrm{kW})}$$

where M = mass flow rate (kgh^−1^), C_p_ = Specific heat (Jkg^−1^K^−1^), and ΔT = inlet–outlet temperature difference (°C).

### 2.5. Electricity Consumption and CO_2_ Concentration

The electricity consumption for heating the control and GHP-installed pig house was calculated every day by installing two separate smart energy electrical submeters for each house. The cost of electricity was calculated by multiplying the electricity consumption by the current electricity cost in South Korea (1 kWh electricity = 39.2 Korean won (KRW) and 39.2 Korean won = 0.033 USD).

The emission of CO_2_ was calculated using the procedure reported by Islam et al. [8] for South Korea. They calculated the CO_2_ emissions as kg CO_2_ equivalent, based on daily electricity consumption, where 1 kWh = 0.547 kg CO_2_ emission equivalent.

### 2.6. Ultrafine Dust (PM_2.5_) and Formaldehyde

The ultrafine dust particulate matter (PM_2.5_) and formaldehyde (FA) levels were determined using a smart sensor air quality meter (AR830A, Dongguan, China) (Supplementary Figure S3) with a range of 0–150 µg/m^3^ for PM_2.5_ and 0–5 ppm for FA, respectively. The concentration of PM_2.5_ and FA was recorded daily at the same time by placing the sensor in the middle of the pig house.

### 2.7. Ammonia (NH_3_) and Hydrogen Sulfide (H_2_S) Concentration

The concentrations of harmful gases (NH_3_ and H_2_S) were calculated by installing a sensor with a range of 0–50 ppm in the middle of the pig house at a height of 1.9 m (Figure 1). The NH_3_ and H_2_S concentrations were determined using a sensoric-NH_3_ 3E 100 SE (City Technology, Bonn, Germany) and using a H_2_S-B4 sensor (Alphasense Ltd., Great Notley, UK), respectively (Supplementary Figure S2).

### 2.8. Growth Performance Measurement

The pigs were weighed individually at the start and end of each period (weaning, growing, and finishing), and the body weight gain (BWG) was recorded. The feed intake (FI) was determined weekly by subtracting the remaining feed from the total feed offered, and the feed conversion ratio (FCR) was calculated.

### 2.9. Statistical Analyses

Statistical analyses were performed using the Statistical Analysis System (SAS, 2011, Version 9.3, Cary, NC, USA). The results are presented as the mean values and the standard error of the mean (SEM). All parameters in the groups were compared by an analysis of variance (ANOVA) and subsequent Duncan’s multiple range test. The effects of the treatment were assessed using the following statistical model (Equation (3)).

Y_ij_ = µ + α_i_ + e_ij_ (3)

where µ = general mean, α_ij_ = effect of treatment (GHP), e_ij_ = random error, and Y_ij_ = response variable. A value of *p* < 0.05 was considered significant.

## 3. Results and Discussion

### 3.1. Pig House Temperature and Relative Humidity

**Figure 4:**
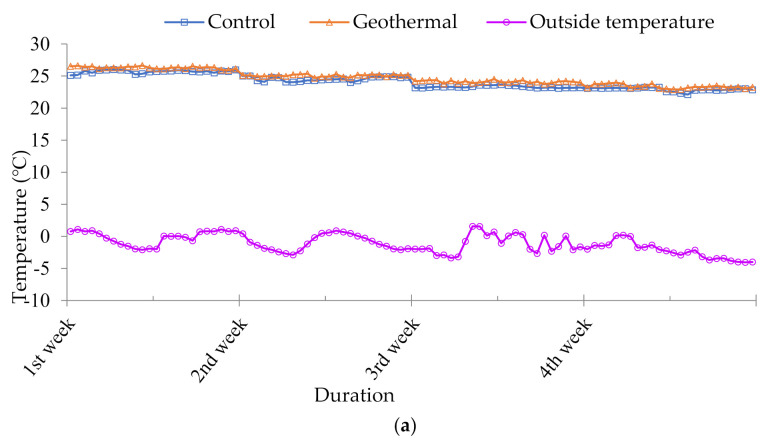

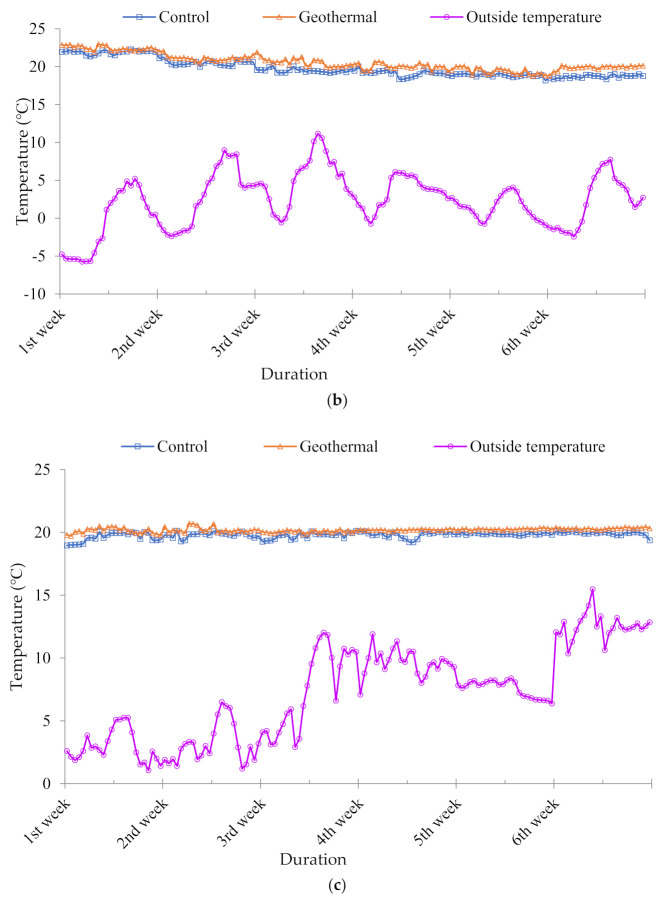
Temperature of the control pig house and geothermal heat pump connected pig house during the experiment. (**a**) Weaning period; (**b**) growing period; (**c**) finishing period.

Figure 4a–c presents the overall distribution pattern of temperature for the control and GHP during each period (weaning, growing, and finishing). The relative humidity was calculated to be 50–60% during the entire experimental duration. The setting temperature was maintained at 26 °C during the first week of the weaning phase then gradually decreased at the rate of 1 °C per week until it reached 20 °C. The 20 °C temperature was maintained until the end of the trial. The mean temperature during each period was significantly higher (*p* < 0.05) in the geothermal heating pig house than the outside temperature. The temperature during weaning was increased by 75% in the GHP system compared to the outside temperature and 7% relative to the control pig house temperature. Similarly, during the growing period, the temperature increased by 80% relative to the outside and 5% relative to the control pig house, while it increased by 83% and 2% compared to the outside and the control pig house temperature, respectively, during the finishing phase.

Environmental conditions can play an important role in pig production, and variation in these parameters may decrease the productivity of pigs. One of the challenging inside-room conditions for pig production is the ambient temperature, which is crucial to the efficiency of pig productivity and growth [20]. Various studies in different countries have reported the heating and cooling efficiency of GHP systems in winter and summer for pig fattening farms, farrowing houses, and broiler houses [2,8,21]. The increased pig house temperature in the GHP system might be due to the efficient heat conversion from groundwater and the uniform distribution by the FCU of the GHP system [8]. The results of the present study are also consistent with the findings of Omer et al. [22], who reported that a GHP could transfer adequate heat from the ground to the source via a hot water storage tank. Furthermore, GHP has a single-loop configuration, which is also called a direct heat exchange system, in which the working fluid of the heat pump passes through the ground heat exchanger and there is no need for a ground loop to heat the pump exchangers, which increases the heating efficiency of the system [22]. In short, a GHP can increase the pig house temperature in the severe cold season.

### 3.2. Coefficient of Performance (COP) and Geothermal Systems Outlet and Inlet Temperature

COP is an important parameter to evaluate the performance and heat efficiency of a heat pump. COP describes how much heat a GHP can generate with every watt of energy consumption [8]. The maximum and minimum COP calculated in this study was 4.10 and 4.50, 4.15 and 4.80, and 4.00 and 5.45 during the weaning, growing, and finishing periods, respectively (Table 2). The mean COP of the GHP system during the 16 weeks was 4.47. The calculated COP of the GHP system is close to the COP of 4.5 and 4.46 calculated for a geothermal heat pump system by Sanner et al. [23] and Islam et al. [8], respectively. The efficiency and performance index of the GHP system was evaluated using the COP. Usually, the COP of the ground source heat pump system (GSHP) ranges from 3.9 to 4.53 in summer and 4.19 to 4.57 in winter [24]. Moreover, GHPs (water–water type) are used widely in Korea owing to their better COP, as calculated in this study, and their ability for both cooling in summer and heating in winter [25].

**Figure 5:**
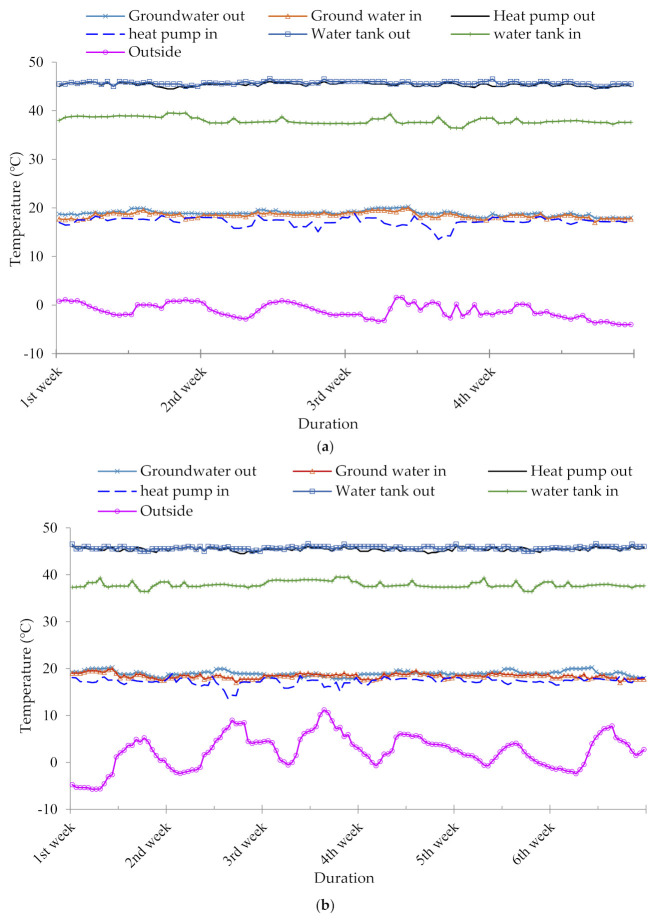

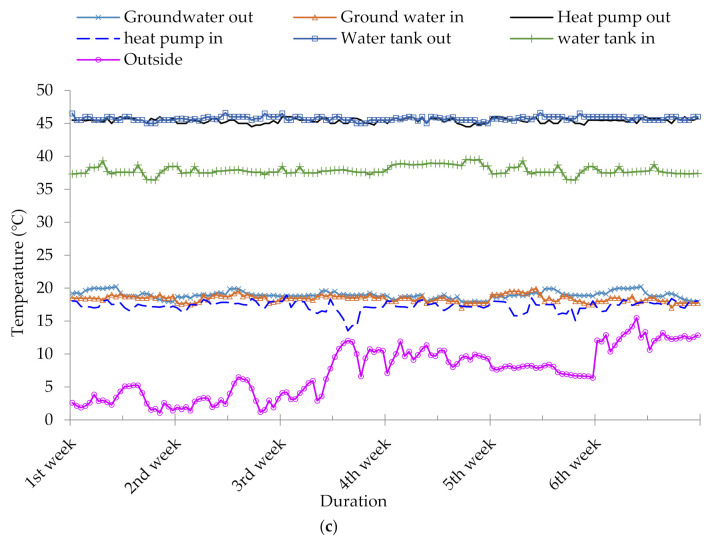
The inflow and outflow water temperature of three pumps of the geothermal system. (**a**) Weaning period; (**b**) growing period; (**c**) finishing period.

The inflow and outflow temperature of the water was examined at three points of the GHP system, including (1) from groundwater to heating pump and vice versa, (2) from heating pump to storage container and vice versa, and (3) from water storage to the pig house and vice versa (Figure 5a–c). The mean difference in the water outflow and inflow temperature was 0.27, 28.90, and 8.20 °C during the weaning phase; 0.25, 27.50, and 7.77 during the growing phase; and 0.20, 25.70, and 7.25 during the finishing phase for the groundwater to heating pump, heating pump to water reservoir, and water storage tank to the pig house, respectively (*p* < 0.05). The increased outflow temperature for the water storage tank and heat pump indicates the efficiency of the GHP system to convert energy to heat and provide sufficient heat to maintain the desired temperature of the pig house throughout the experiment.

### 3.3. Formaldehyde, Ultrafine Dust (PM_2.5_), NH_3_, and H_2_S Concentration

The concentration of H_2_S gas and PM_2.5_ in the pig house was unaffected (*p* > 0.05) by the heating system during the experimental period (Table 3 and Table 4). On the other hand, NH_3_ concentrations were lowered significantly (*p* < 0.05) during the growing and finishing period in the GHP house compared to the control pig house (Table 2). Furthermore, the formaldehyde content was decreased significantly (*p* < 0.05) in the GHP house during the weaning period (Table 3).

The release of noxious gases from the animal houses, such as NH_3_ and H_2_S, has harmful effects on the animal health and production, environment, and health of workers staying at the farms [26]. The release of NH_3_ also depends on the climate conditions of the house, and concentration is positively correlated with the ventilation rate and ambient temperature [5]. Similarly, the concentrations of PM_2.5_ and formaldehyde from the pig house have adverse effects on the respiratory health of the animals and humans as well as a negative impact on the environment [27]. Misselbrook et al. [28] reported that the daily housing emission factor of 79.2 for fattening pigs per livestock unit (LU is equivalent to 500 kg live weight). Furthermore, Liu et al. [29] calculated that the median emission rates of H_2_S and NH_3_ from pig houses were 0.09 and 2.78 kg/y, respectively. In the present study, there was an average 48% decrease in the concentration of NH_3_ from the GHP-equipped pig house compared to the control. The reason for the reduced NH_3_ concentration can be the improved air quality in the GHP pig house because GHP provides fresh air continuously and dilutes the NH_3_ inside the pig house [30]. The results of this experiment are consistent with Jacobson et al. [31], who reported that a GHP system reduced the concentration of NH_3_ by 20–30% when heating the farrowing house. Pulat et al. [32] also described geothermal energy as a renewable and eco-friendly energy source that plays an important role in reducing global climate change, enhancing energy security, and protecting public health. On the other hand, a small amount of noncondensable gases, such as CO_2_, NH_3_, H_2_S, methane (CH_4_), and nitrogen (N_2_), may be emitted through the GHP system, which is harmless to the animals and workers [33].

Ultrafine dust has become an emerging major public health concern in South Korea. PM_2.5_ can penetrate the lungs, placental barrier, and blood–brain barrier [34]. Although no significant differences in the levels of ultrafine dust were observed between the control and GHP system, the values of PM_2.5_ were in accordance with the standards of national ambient air quality standards (AQS) in Korea. The AQS of PM_2.5_ was 50 µg/m^3^ for 24 h [34]. Formaldehyde is an eye and skin irritant and is also classified as a carcinogenic agent. One of the primary sources of formaldehyde emission into the environment is heating systems and waste incinerators [35]. The GHP system reduced the concentration of formaldehyde significantly in the weaning phase, which requires more heating compared to other periods of pig production. Formaldehyde at concentrations of 0.10–0.11 ppm cause symptoms in both animals and humans [35]. In the present study, the average formaldehyde level in the GHP pig house was in the acceptable range as recommended.

### 3.4. Electricity Consumption, CO_2_ Concentration, and Cost

Table 5 lists the electricity consumption and CO_2_ concentration of the GHP-installed and conventional electricity-heated pig house. During the entire experimental trial, the electricity consumption by the GHP system was 38% lower (by 2743 kWh) than the conventional system. This might be caused by the efficient and uniform heat distribution by the GHP system because of its high COP and fewer operating hours. Charoenvisal [12] also reported that GHP could supply three units of electricity per unit consumption, which can eventually decrease the consumption of electricity.

Electricity production comprises the second-largest percentage (26.9%) of greenhouse gas emissions (GHE), which leads to a range of health and environmental effects [36]. Therefore, there is a high demand for alternative energy sources to reduce environmental pollution. In the present study, CO_2_ emissions were reduced significantly (by 1501 kg) in the GHP system relative to the control. GHP can decrease the GHE substantially, which is important for not only the environment but also for the well-being of workers and animals [31]. In support of these findings, many scientists [8,17,19] have reported that CO_2_ concentration and energy consumption were reduced by installing GHP systems for heating buildings and animal farms because of their energy efficiency.

**Figure 6:**
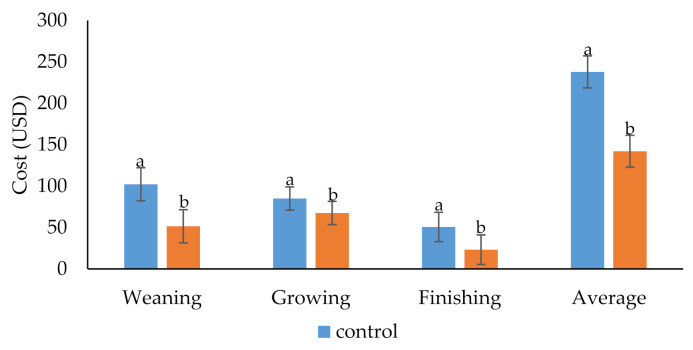
The effect of the geothermal heat pump (GHP) on the cost of electricity during the weaning, growing, and finishing period. a,b Bars at a particular point with different alphabet differ significantly (*p* < 0.05).

Figure 6 shows the heating cost of the GHP-supported pig house and the control. The electricity expenditure for the GHP system was reduced significantly (by 40%) compared to the conventional heating system. Similarly, Barbier et al. [17] reported a decrease in the cost of electricity using a GHP for heating industrial and agricultural facilities. GHP systems use a renewable energy source, whereas conventional heating uses the combustion of fuels to generate electricity and heating. This might explain the low electricity cost in a GHP-installed pig house. The cost of heating pig houses is one of the major challenges for pig farmers because the price of fuel and electricity is increasing continuously in Korea and throughout the world. In the present study, GHP was more economical than the control under severe weather conditions. Moreover, the energy produced by the GHP decreased the GHE concentration in the environment, which finally decreased environmental pollution [15]. Therefore, the ground source geothermal heat pump is an effective renewable, alternative, and environmentally friendly energy source in Korea.

### 3.5. Production Performance Parameters

Table 6 lists the effects of the GHP on the production performance parameters of pigs. The BWG, FI, and FCR did not differ (*p* > 0.05) between the control and GHP-equipped pig house.

Growth performance parameters, such as the market weight and FCR, are important economic parameters for swine farmers in pig production [37]. These attributes must be considered first before any technology can be introduced in pig farming. In the present study, the GHP system had no negative effect on pig performance compared to the conventional heating system. These results are in agreement with Choi et al. [16], who reported that the geothermal heating pump system (GHPS) did not affect the BWG and FI in the farrowing house. Moreover, the production performance is a multifactorial dependent parameter that not only depends on the environmental conditions but also the management, feed, disease incidence, and genetic potential [38].

### 3.6. Annual Operational and Initial Installation Cost

Table 7 lists the initial installation and operational cost for the GHP and conventional system. Despite the higher initial investment for the GHP system, the operational expense was 50% lower than the conventional system because of energy efficiency and a higher COP. In the present study, GHP was installed at a research farm, which is relatively smaller than the commercial pig farm. Therefore, the operational cost and profit margin may be improved further at a commercial farm. As a result of rapid economic development, many countries are facing a huge challenge in energy shortage, environmental pollution, and climate change. Energy shortage is one of the most serious problems, and renewable energy reduces the dependence of food production on fossil fuels as well as decreases GHE. Therefore, the combination of food production with renewable energy would be a prosperous implementation to increase food security by enhancing production at a lower cost [39]. 

Thus, the GHP system is likely to be accepted in the future considering the heating effect, energy-saving, welfare and health of animals and workers, and environmental pollution. On the other hand, the assistance of governments through subsidies will be necessary to broaden the installation of GHP systems to animal farms for energy-saving and protecting the environment [40].

## 4. Conclusions

The GHP system reduced energy consumption and electricity cost during the experimental period. The CO_2_ emission and noxious gas (NH_3_ and formaldehyde) emissions were also reduced in the GHP-installed pig house. Despite the high initial installation cost, pig producers could benefit in the long term in commercial production with lower operational costs and reduced environmental pollution. Therefore, the GHP system might be a more effective sustainable renewable energy source in the future for pig performance and welfare, cost-saving, and mitigating environmental pollution.

## Figures and Tables

**Table 1:** Ingredients and chemical composition of the experimental diets.

	Weaning Pigs, 0–4 Weeks	Growing Pigs, 4–10 Weeks	Finishing Pigs, 10–16 Weeks
Ingredients (% as fed basis)			
Yellow corn	47.80	51.36	55.00
Rice bran	14.00	7.00	8.00
Rapeseed oil	-	1.72	3.00
DDGS ^1^	-	6.00	6.00
Soybean meal	22.10	21.80	18.16
Limestone	0.70	0.84	1.00
Calcium phosphate	0.70	0.10	0.20
Salt	0.15	0.30	0.30
Vit-min premix ^2^	0.50	0.45	0.20
Animal fat	7.00	6.78	4.76
Molasses	2.00	2.50	2.50
Amino acid additive	5.05	1.15	0.88
Chemical composition (% DM)			
ME ^3^ (kcal/kg)	3350.00	3265.00	3265.00
Crude protein	19.00	18.00	17.00
Ca (%)	0.70	0.80	0.80
Available P (%)	0.44	0.34	0.34
Lysine (%)	1.35	1.20	1.10
Methionine (%)	0.53	0.37	0.31

^1^ Distiller’s dried grains with solubles. ^2^ Vit-min premix provided following nutrients per kg of premix: vitamin A, 6000 IU; vitamin D_3_, 800 IU; vitamin E, 20 IU; vitamin K_3_, 2 mg; thiamin, 2 mg; riboflavin, 4 mg; vitamin B_6_, 2 mg; vitamin B_12_, 1 mg; pantothenic acid, 11 mg; niacin, 10 mg; biotin, 0.02 mg; CU—copper sulfate, 21 mg; Fe—ferrous sulfate, 100 mg; Zn—zinc sulfate), 60 mg; Mn—manganese sulfate, 90 mg; I—calcium iodate, 1.0 mg; CO—cobalt nitrate, 0.3 mg; Se—sodium selenite, 0.3 mg. ^3^ Metabolizable energy.

**Table 2:** Coefficient of performance (COP) of the geothermal heat pump (GHP) during heating.

Periods	Heat Pump Water Flow Temperature (°C)		Difference	Heat Pump Consumption (kWh)	COP
	Outflow	Inflow			
Weaning					
1st week	44.11	17.55	26.56	44.80	4.10
2nd week	44.22	14.96	29.26	44.20	4.46
3rd week	43.92	11.75	32.17	48.20	4.50
4th week	44.16	15.30	28.86	44.60	4.40
Growing					
1st week	44.47	17.90	26.57	44.40	4.15
2nd week	44.15	16.60	27.55	39.70	4.64
3rd week	44.16	17.30	26.86	41.00	4.45
4th week	44.04	12.75	31.29	43.00	4.80
5th week	44.34	15.65	28.69	44.40	4.40
6th week	44.20	17.40	26.80	37.90	4.70
Finishing					
1st week	44.50	18.30	26.20	43.10	4.20
2nd week	44.45	18.45	26.00	41.50	4.30
3rd week	44.15	18.65	25.50	45.50	4.00
4th week	43.85	18.92	24.93	29.40	5.45
5th week	44.04	19.00	25.04	36.50	4.60
6th week	44.05	17.90	26.15	37.50	4.65
Average	44.00	16.75	27.25	44.50	4.47

**Table 3:** Effect of the geothermal heat pump (GHP) on ammonia (NH_3_) and hydrogen sulfide (H_2_S) concentrations in the pig house (ppm).

Items	Control	GHP	SEM ^1^	*p*-Value
NH_3_ emission				
Weaning	0.00	0.00	0.00	-
Growing	0.16 ^a^	0.13 ^b^	0.18	<0.0001
Finishing	0.97 ^a^	0.46 ^b^	0.35	<0.0001
Average	0.42 ^a^	0.22 ^b^	0.37	<0.0001
H_2_S emission				
Weaning	0.00	0.00	0.00	-
Growing	0.00	0.00	0.00	-
Finishing	5.14	5.35	0.45	0.9034
Average	1.93	2.01	0.67	0.9042

^a,b^ Values with different superscripts differ significantly. ^1^ Standard error of the mean.

**Table 4:** Effect of the geothermal heat pump (GHP) on formaldehyde (ppm) and ultrafine dust (PM_2.5_) (µg/m^3^) in the pig house.

Items	Control	GHP	SEM ^1^	*p*-Value
Formaldehyde				
Weaning	0.06 ^a^	0.04 ^b^	0.03	0.0436
Growing	0.08	0.05	0.01	0.0649
Finishing	0.13	0.14	0.03	0.8146
Average	0.10	0.09	0.12	0.5955
Ultrafine dust (PM_2.5_)				
Weaning	28.39	27.22	17.76	0.8242
Growing	29.03	27.48	28.83	0.7932
Finishing	21.14	21.20	14.72	0.9871
Average	25.84	25.00	19.31	0.7709

^a,b^ Values with different superscripts differ significantly. ^1^ Standard error of the mean.

**Table 5:** Effect of the geothermal heat pump (GHP) on electricity consumption (kWh) and CO_2_ concentration (kg) in the pig house for 16 weeks.

Periods	Electricity Use		Reduced	CO_2_ Emission		Reduced
	Control	GHP		Control	GHP	
Weaning	3053 ^a^	1565 ^b^	1488	1670 ^a^	856 ^b^	814
Growing	2524 ^a^	2035 ^b^	507	1390 ^a^	1113 ^b^	277
Finishing	1467 ^a^	719 ^b^	748	803 ^a^	393 ^b^	410
Total	7062 ^a^	4319 ^b^	2743	3863 ^a^	2362 ^b^	1501

^a,b^ Values with different superscripts differ significantly.

**Table 6:** Effect of the geothermal heat pump (GHP) on production performance parameters.

Item	Control	GHP	SEM ^1^	*p*-Value
Weaning period (0–4 weeks)				
Initial weight (kg)	8.56	8.89	2.87	0.8158
Final weight (kg)	25.81	26.81	4.48	0.6506
Weight gain (kg)	17.26	17.92	2.38	0.5627
Feed intake (kg)	33.48	36.09	6.19	0.3945
FCR (feed/gain)	1.95	2.03	0.37	0.6448
Growing period (4–10 weeks)				
Initial weight (kg)	25.81	26.81	4.48	0.6506
Final weight (kg)	70.77	68.53	5.47	0.4313
Weight gain (kg)	44.96	41.72	3.29	0.0726
Feed intake (kg)	100.93	105.44	14.59	0.5216
FCR (feed/gain)	2.25	2.53	0.33	0.0975
Finishing period (10–16 weeks)				
Initial weight (kg)	70.77	68.53	5.47	0.4313
Final weight (kg)	113.43	108.59	7.24	0.1801
Weight gain (kg)	42.66	40.06	4.39	0.2449
Feed intake (kg)	150.81	143.46	21.28	0.4801
FCR (feed/gain)	3.53	3.58	0.32	0.7533
Average period (0–16 weeks)				
Initial weight (kg)	8.56	8.89	2.87	0.8158
Final weight (kg)	113.43	108.59	7.24	0.1801
Weight gain (kg)	104.88	99.70	1.83	0.0900
Feed intake (kg)	285.23	284.99	11.34	0.9895
FCR (feed/gain)	2.71	2.85	0.08	0.2686

^1^ Standard error of the mean.

**Table 7:** Operational and installation cost of the geothermal heat pump (GHP) and a conventional heating system.

	Conventional Heating	GHP	
Installation cost (USD)	4420	17,676	According to the manufacturer’s instruction
Life span	Up to 5 years	(1) Ground loop, 50 years	
		(2) Heat pump, 25 years	
Operational cost annually ^1^ (USD)	713.52	426.12	
Depreciation time	5 years	25 years	

^1^ Annual operational cost was calculated based on the yearly electricity consumption and local unit price.

## Supplementary Materials

The following are available online at https://www.mdpi.com/2076-2615/10/11/2075/s1, Figure S1: A geothermal heat pump system, Figure S2: NR-TH set for temperature and humidity detection. Sensors for determination of NH_3_ and H_2_S emission, Figure S3: Smart sensor air quality meter, Table S1: Specifications of geothermal heat pump, Table S2: Specifications of exhaust fans in pig house.

## Nomenclature

ANOVA	Analysis of variance
AQS	Ambient air quality standards
BHE	Borehole exchanger
BWG	Body weight gain
CH_4_	Methane
COP	Coefficient of performance
CO_2_	Carbon dioxide
C_p_	Specific heat (kcal/kg °C)
FA	Formaldehyde
FCR	Feed conversion ratio
FCU	Fan coil unit
FI	Feed intake
GER	Geothermal energy resources
GHP	Geothermal heat pump
HPU	Heating pump unit
H_2_S	Hydrogen sulfide
KRW	Korean won
kW	Kilowatt
kWh	kilowatt-hour
M	mass flow rate (kg/h)
m	meters
NH_3_	Ammonia
N_2_	Nitrogen
PM_2.5_	Particulate matter
SEM	Standard error of the mean
ΔT	Inlet–outlet temperature difference (°C)
µ	General mean
α_ij_	Effect of treatment
e_ij_	Random error
Y_ij_	Response variable
