# Supplementary material for: Effect of a Heating System Using a Ground Source Geothermal Heat Pump on Production Performance, Energy-Saving and Housing Environment of Pigs
Source: Animals (Basel). 2020 Nov 9;10(11):2075. doi: 10.3390/ani10112075 (PMC7695292; doi:10.3390/ani10112075)
Supplement: Supplementary file 1 [file animals-10-02075-s001.pdf]

Supplementary file

Effect of a heating system using a geothermal heat pump on production performance, energy saving and housing environment of pigs

Supplementary Table 1. Specifications of geothermal heat pump.

Certification Number	GT-WWH-1-0170	Installation name	Water-water geothermal heat pump unit
Model name	DHGW 5N-C4-02	Manufacture company	Daesung Heat Enersys
Rating consumption electric power	4.93 kW	Rating consumption current	8.99 A
Internal pressure test	High pressure section 4.07 MPa<br>Low pressure section 2.42 MPa	Max current consumption	16.7 A
Maximum working pressure	High pressure section 3.7 MPa<br>Low pressure section 2.2 MPa	Power supply	Three-phase four-wire system<br>380 V 60 Hz
Refrigerant name	R-410A	Legal freezing capacity	2.1 RT
Refrigerant injection amount	1.35 kg	Product weight	115 kg
Inspection passed heat Source	2014. 08.	Manufacturing number	PGW005140680
Date of manufacture	2014. 08.		
Heating ability	IW40-W10 / B05 21.97 / 19.66 kW (5.59 USRT)		
Cooling ability	IW12-W15 / B25 21.86 / 20.59 kW (5.85 USRT)		

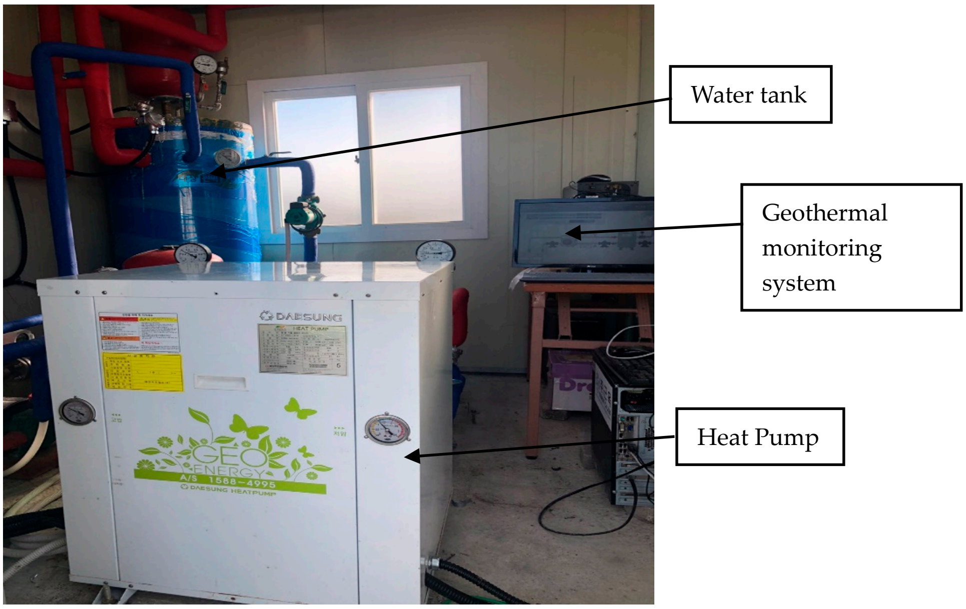

Supplementary Figure 1. A geothermal heat pump system.

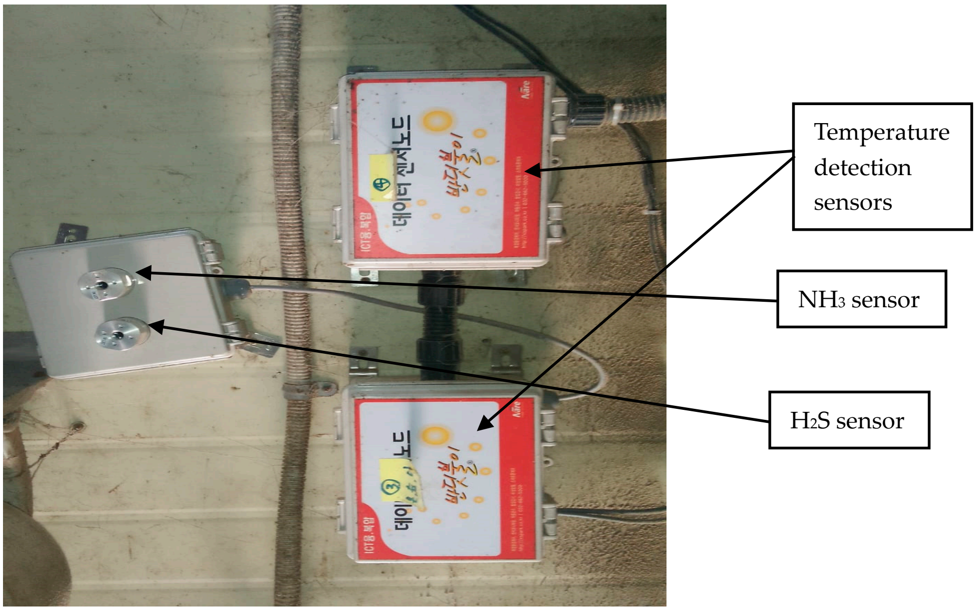

Supplementary Figure 2. NR-TH set for temperature and humidity detection. Sensors for determination of NH_3 and H_2S emission.

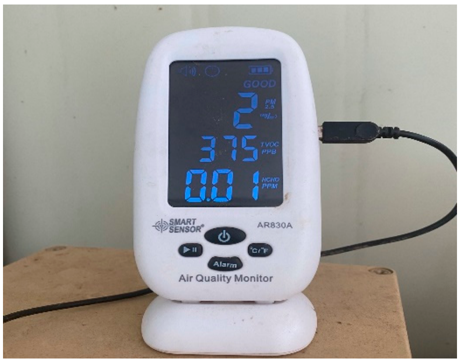

Supplementary Figure 3. Smart sensor air quality meter.

Supplementary Table 2. Specifications of exhaust fans in pig house.

Fan[1]	Model name	HP (Horse Power)	RPM (1 minute)	Ventilatiory volume (CFM)	
				10%	100%
Big fan	KA-400	0.1	1,150	677	1,574
Small fan	KA-300	0.1	1,700	451	1,097

[1] Each big house has 1 big fan and 2 small fans.
